# Long-read DNA and RNA sequencing reveal an intronic retrotransposon insertion in *TCOF1* causing Treacher Collins syndrome

**DOI:** 10.1016/j.xhgg.2025.100523

**Published:** 2025-09-27

**Authors:** Federico Ferraro, Nikolas Kühn, Dmitrijs Rots, Herma C. van der Linde, Banin Mohseni, Leontine van Unen, Mark Drost, Mark Nellist, Marieke Koekkoek, Rachel Schot, Henriette W. de Gier, Mieke Pleumeekers, Tahsin Stefan Barakat, Tjitske Kleefstra, Marjolein Weerts, Marieke F. van Dooren, Tjakko J. van Ham

**Affiliations:** 1 Department of Clinical Genetics, Erasmus University Medical Center, Rotterdam, the Netherlands; 2 Department of Otorhinolaryngology, Sophia Children’s Hospital, Erasmus University Medical Center, Rotterdam, the Netherlands; 3 Department of Plastic Surgery, Sophia Children’s Hospital, Erasmus University Medical Center, Rotterdam, the Netherlands

**Keywords:** RNA sequencing, long-read genome sequencing, Treacher Collins syndrome, retrotransposon insertion

## Abstract

Treacher Collins syndrome (TCS) is a craniofacial genetic disorder caused by loss-of-function variants in *TCOF1*, *POLR1B*, *POLR1C*, or *POLR1D*. Here, we describe two previously undiagnosed paternal half-siblings affected with clinical TCS, and their apparently unaffected father. Diagnostic short-read RNA sequencing) identified aberrant expression of *TCOF1* and optical genome mapping detected a large genomic insertion therein. Long-read genome sequencing (lrGS) resolved a deep intronic 3.5 kb SINE-VNTR-Alu (SVA) retrotransposon insertion in intron 17 of *TCOF1*. Long-read RNA sequencing (lrRNA-seq) demonstrated that the insertion was partially exonized inducing isoform switch to the shorter non-canonical *TCOF1* isoform c. SVA insertion was confirmed in both half-siblings, and we detected mosaicism in the father. This work demonstrates the potential of lrRNA-seq and lrGS, to identify pathogenic variants in unexplained genetic disorders.

## Teaser

We report two paternal half-siblings affected by Treacher Collins syndrome, caused by a pathogenic retrotransposon insertion in *TCOF1*. We show the utility of short-read RNA sequencing to prioritize the affected gene, long-read genome sequencing to resolve the event, and long-read RNA sequencing to characterize the functional impact of the insertion.

## Introduction

Treacher Collins syndrome (TCS) (MIM: 154500) is a rare congenital genetic disorder affecting craniofacial development caused in 89% of the patients by pathogenic variant in *TCOF1* (OMIM: 606847).^1^ *TCOF1* encodes a nucleolar protein that plays a crucial role in the synthesis of ribosomal RNA (rRNA) and proper craniofacial development.^1^ *TCOF1* haploinsufficiency leads to cell-autonomous defects in neural crest cell formation and proliferation.^2^ To date, over 200 autosomal dominant (AD) pathogenic variants in *TCOF1* have been reported,^1^ typically leading to the production of a truncated and/or non-functional protein. In addition to pathogenic AD variants in *TCOF1*, a minority of TCS individuals result from pathogenic variants in *POLR1B* (AD), or pathogenic autosomal recessive variants (AR) in *POLR1C* or *POLR1D* (AD or AR). These genes encode subunits of RNA-polymerase I, which is required for rDNA transcription, converging on the same mechanism as *TCOF1*. It is estimated that in ∼4% of typical TCS a molecular cause cannot be identified, suggesting either the presence of a pathogenic variant in one of the known genes undetected by current technology, or possibly in another gene not currently linked to TCS.^3^

Here, we report an unconventional molecular genetic mechanism causing *TCOF1* haploinsufficiency in two half-siblings with a clinical diagnosis of TCS. After a diagnostic odyssey of almost a decade, multi-omics investigations finally resolved a SINE-VNTR-Alu (SVA) retrotransposon insertion in intron 17 of *TCOF1* leading to impaired production of the canonical *TCOF1* isoform. We confirmed the presence of the insertion in both affected individuals, and the inheritance from their mosaic healthy father. We describe a type of variant not previously found in TCS and a novel pathogenic mechanism, emphasizing the utility of combined technologies to identify and interpret complex pathogenic variants currently missed by short-read exome sequencing.

## Materials and methods

### Ethics approval

All subjects or legal guardians provided written informed consent for publication of this manuscript. Specific informed consent was obtained for the publication of clinical pictures. The study was conducted in accordance with the 1984 Declaration of Helsinki and its subsequent revisions.

Individuals were examined at Erasmus MC (Rotterdam, the Netherlands). Informed consent for diagnostic tests, research investigations, to share photographic, clinical, and analysis data was obtained. Chromosomal microarray and exome sequencing (ES) were performed following standard operating procedures.^4^ Use of genome-wide technologies for diagnostic purposes was previously approved (Institutional Review Board MEC-2012-387).

Short-read RNA sequencing (srRNA-seq) and outlier analysis was performed as recently described.^5^ For nanopore direct long-read RNA-seq, RNA was performed following the Oxford Nanopore Direct RNA sequencing (SQK-RNA004) protocol.

Genomic DNA was extracted following standard operating procedures. OGM was conducted as described previously.^6^ For lrGS, gDNA was processed following the Oxford Nanopore Ligation Sequencing Kit (SQK-LSK114). Basecalling and alignment to the reference genome were performed in MinKNOW v23.07.12 using the super accuracy (SUP) model. Structural variants were called by sniffles2.^7^ The SVA insertion was annotated with RepeatMasker v.4.1.5.^8^

For variant confirmation and segregation, we performed allele-specific PCR. Briefly, a universal forward primer (5′-TCTCCACAACAGCCCTATGA-3′) was used in combination with a reverse primer specific for the allele with (5′-GAGACGGGACCTTTTCTGC-3′) or without the insertion (5′-AAAAATCTCTCAATCAGGAAGAGG-3′) with expected product sizes of 420 and 498 bp, respectively. PCR products were visualized on agarose gel to determine the presence of either allele or, where applicable, used for Sanger sequencing.

## Results

**Figure 1:**
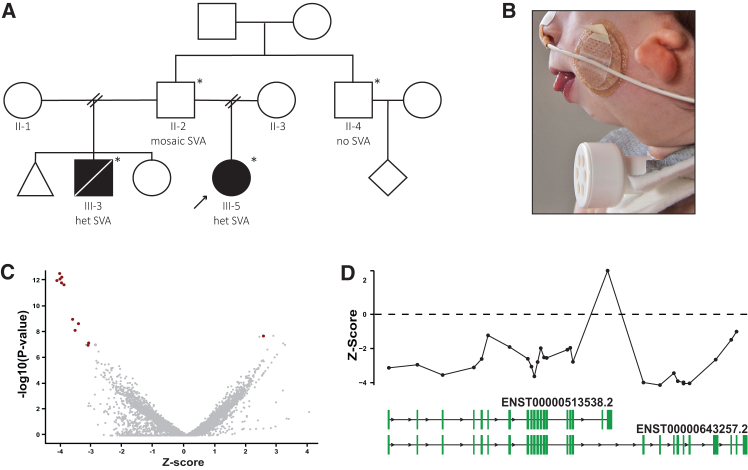
Familial history and srRNA-seq outlier analysis identifying *TCOF1* aberrant expression (A) Pedigree including the affected individuals. Black arrow highlights the index of this study; asterisks indicate individuals whose DNA was available for the investigation. (B) Photograph of III-3. Note severe mandibular hypoplasia/retrognathia, microtia, and tracheostomy. (C) Exon-level volcano plot showing the relative decreased expression of most of the *TCOF1* exons and upregulation of one of them (belonging to the isoform c) (red) vs. other genes’ exons (gray). *p* value and *Z* score calculated according to Dekker et al.^5^ (D) *Z* score plot showing the relative expression level of the exons of *TCOF1* long and short isoforms, drawn below.

We were referred a 1-year-old boy (III-3) presenting with a clinical TCS phenotype, including mandibular and maxillary hypoplasia, microtia and hearing loss, down-slanted palpebral fissures, cleft palate, and obstructive airway problems, requiring several surgical procedures (Figures 1A and 1B). Routine diagnostics consisting of ES and chromosomal microarray analysis did not reveal pathogenic variants in *TCOF1*, *POLR1B*, *POLR1C*, or *POLR1D*, or in other Mendelian disease genes. The boy remained genetically undiagnosed and passed away at 4 years of age after a choking incident.

**Figure 2:**
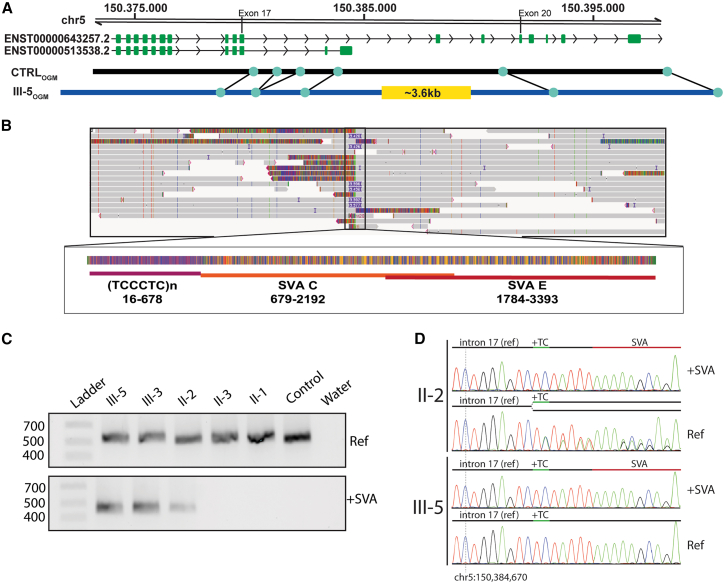
Genomic identification of an intronic SVA insertion in *TCOF1* causing TCS (A) Schematic representation of the optical genome mapping results. (B) RepeatMasker annotation results of the intronic SVA insertion. (C) Gel electrophoresis of the allele-specific PCR products confirming the SVA insertion and showing its segregation in the pedigree. (D) Sanger sequencing results of the allele-specific PCR products.

Five years after the examination of III-3, an infant paternal half-sister (III-5), also with a clinical presentation strongly suggestive of TCS, was referred to us (Figure 1A). However, molecular genetic investigations as in III-3 were inconclusive. Subsequent srRNA-seq outlier analysis in fibroblasts^5^ from III-5 indicated reduced expression of the canonical *TCOF1* transcript (Ensembl: ENST00000643257.2) and apparent upregulation of a minor *TCOF1* transcript, isoform c (Ensembl: ENST00000513538.2) (Figures 1C and 1D).^9^ Manual inspection of four heterozygous *TCOF1* SNPs detected by srRNA-seq showed slightly skewed expression, with one allele being expressed at an average allele frequency of 0.33, which increased to 0.39 for fibroblasts treated with cycloheximide. Neither re-inspection of ES data nor targeted PCR and Sanger sequencing analysis identified variants that could explain the abnormal *TCOF1* expression. OGM of fibroblast DNA of III-5 suggested a heterozygous ∼3,600 bp insertion between exons 17 and 20 of the canonical *TCOF1* transcript but could not resolve the insertion precisely (Figure 2A).

Therefore, we performed nanopore long-read genome sequencing (lrGS) in DNA isolated from blood of III-5 and identified a heterozygous 3,396 bp (chr5(GRCh38):g.150384687_150384688ins3396, NM_001371623.1(TCOF1):c.2860-3215_2860-3214insN3396; p.?) insertion (Clinvar ID: SCV007126586) in intron 17 of the canonical *TCOF1* isoform, just downstream of the minor *TCOF1* isoform c (Figure 2B), unobserved in 48 unrelated in-house long-read genomes. The inserted sequence consisted of a (1) hexamer repeat (TCCCTC)n (nucleotides 16–678), (2) a retrotransposon of class SVA C (nucleotides 679–2,192), and (3) a partially overlapping SVA E (nucleotides 1,784–3,393) (Figure 2B). The hexamer repeat was determined to be part of the SVA C, as described previously.^10^ The 15 nucleotides upstream likely represent target site duplication, a characteristic hallmark of retrotransposon insertion.^11^

We confirmed the insertion in DNA isolated from blood in both half-siblings and their father with allele-specific PCR, agarose gel electrophoresis, and Sanger sequencing (Figures 2C and 2D). Sanger sequencing of the PCR product without SVA from the father revealed the presence of two alleles therein, marked by the heterozygous polymorphism chr5:150384675-G-GTC (gnomAD v.4.1.0 minor allele frequency 60%) (Figure 2D). This suggests that the father is a gonadosomatic mosaic carrier of the SVA, explaining how he could transmit a pathogenic *TCOF1* variant while clinically unaffected.

**Figure 3:**
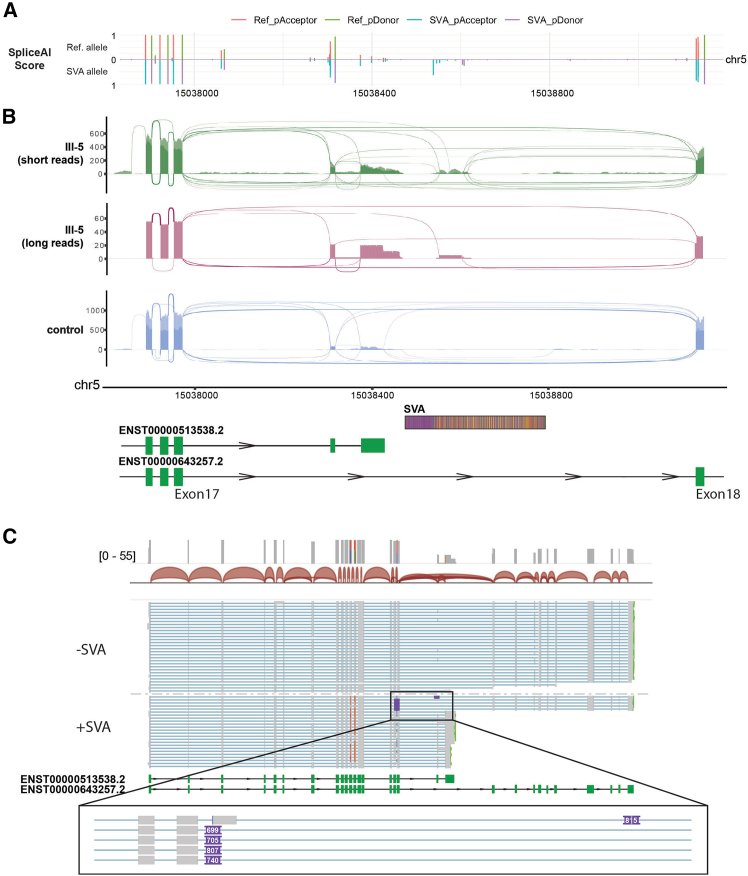
Functional characterization of the intronic SVA in TCOF1 (A) SpliceAI predicted scores on reference allele and allele containing the SVA sequence. (B) ggsashimi plot of the aligned srRNA- and long-read RNA sequencing (lrRNA-seq) data from III-5 and unrelated control on a reference genome containing the SVA sequence as detected in III-5. For short-read data, two overlapping tracks are provided; light color is CHX− cells, darker color CHX+ cells. Schematics of the transcripts and of the SVA are aligned below the ggsashimi. (C) IGV snapshot of the phased direct lrRNA-seq of fibroblasts from III-5.

We then investigated how the SVA insertion could explain the isoform switch inferred from the srRNA-seq data. SpliceAI^12^ predicted donor and acceptor sites within the SVA suggesting its possible exonization (Figure 3A). To investigate this, we included the insertion in the reference genome and realigned the srRNA-seq data to the bespoke reference sequence. In both cycloheximide-treated and untreated cells, we detected the expression and incorporation of ∼750 bp of the SVA C together with *TCOF1* exons (Figure 2B), in correspondence to the sites predicted by SpliceAI (Figure 3A), confirming the SVA exonization, already described for other pathogenic SVA insertions.^13^ Finally, to completely resolve the impact of the insertion on *TCOF1* transcripts, we performed long-read RNA-seq of native RNA from cycloheximide-treated fibroblasts of III-5 (Figure 3B). We phased the reads and observed that, while most of the reads from the wild-type allele represented the canonical isoform of *TCOF1* (ratio 29:1 long over short), the allele containing the SVA was preferentially represented by the isoform c (ratio 5:19 long over short) (Figure 3C). Moreover, the five reads representing the long isoform from the SVA-containing allele always contained exonized portions of the SVA. This was consistent with the srRNA-seq data and highlighted a complex splicing pattern involving different exons and the SVA insertion*. In silico* translation of the reads containing the SVA revealed a frameshift and the introduction of a termination codon within the exonized SVA sequence likely to cause nonsense-mediated mRNA decay,^14^ in line with the observation from srRNA-seq from fibroblasts untreated and treated with cycloheximide.

## Discussion

We describe two half-siblings with TCS, caused by the insertion of ∼3.4 kb SVA-type retrotransposon into intron 17 of *TCOF1* transmitted by their mosaic unaffected father. Noteworthy, while pathogenic SVA insertions previously reported in other disorders belong to the classes E and F,^15^ here we report a hybrid E/C-class SVA associated with a genetic disease.

Multi-omics investigations were crucial in detecting *TCOF1* expression disruption, the pathogenic intronic SVA insertion, and in clarifying its impact. Given that the predicted protein product of the short isoform lacks a nucleolar localization signal,^9^ it seems likely that a switch to this isoform would abolish the role of TCOF1 in ribosome biosynthesis, converging on the same molecular mechanism as other TCS-causing variants, so far only reported for the long canonical isoform.

The accurate mapping and identification of transposable element insertions remain a significant challenge both for srGS and for srRNA-seq.^16^ Specific tools aimed at addressing these challenges are emerging and, while they are not yet established for diagnostic purposes, they will likely enhance the diagnostic yield by detecting similar events while also determining their consequences at the mRNA level. Our results highlight that long-read sequencing technologies can also help bridge this gap: lrGS was pivotal in characterizing at base-pair resolution the insertion, whereas long-read RNA sequencing captured full-length aberrant *TCOF1* isoforms, predicted the early stop codon, and phased the reads, providing hints for the potential pathogenic mechanism.

Retrotransposition has been increasingly recognized as a causative event in genetic diseases.^17^^,^^18^ Given that *de novo* transposition events have been estimated to occur at rates of up to 1:20 live births for just L1 elements alone,^19^ retrotransposon-mediated gene disruption could be more common than currently recognized due to technological limitations.

Strikingly, both half-siblings inherited the SVA insertion from their healthy father, who carries the insertion in mosaic form, suggesting that the SVA activation and retrotranscription occurred during early embryonic development and is not limited to only gametogenesis. Interestingly, a family with a similar inheritance pattern but with a pathogenic frameshift variant was reported recently.^20^ Given that TCS is considered to exhibit incomplete penetrance and 60% of cases are thought to result from *de novo* mutations, we suspect that there are more mosaic parental carriers that account for part of this.

In summary, by combining several innovative molecular diagnostic techniques, we identified an intronic SVA insertion disrupting *TCOF1* in a family with TCS. In addition to providing the family with a molecular diagnosis, in this case understanding the inheritance was also directly relevant for the paternal siblings’ reproductive choices. Our results demonstrate the potential added value of combining DNA and RNA-based long-read approaches for complete resolution of complex variants and of their effects.

## Data and code availability

There are restrictions regarding the availability of underlying genetic data due to existing privacy regulations.

## Acknowledgments

We thank the patients and families for their participation. This research was supported by funding from the Netherlands Organization for Health Research and Development (ZonMw) (grant no. 91718310 to T.K.).

## Author contributions

Conceptualization, F.F., M.D., M.F.v.D., and T.J.v.H.; methodology, F.F., M.N., D.R., L.v.U., B.M., H.C.v.d.L., R.S., and M.K.; formal analysis and investigation, all authors; writing – original draft, N.K., F.F., M.D., and T.J.v.H.; writing – review & editing, N.K., M.D., F.F., D.R., H.C.v.d.L., T.S.B., M.N., T.K., M.W., M.F.v.D., and T.J.v.H.; supervision, T.K. and T.J.v.H. All authors read, commented, and approved the final manuscript.

## Declaration of interests

The authors declare no competing interests.

## Web resources

OMIM, http://www.omim.org/
